# Differential Adhesion between Moving Particles as a Mechanism for the Evolution of Social Groups

**DOI:** 10.1371/journal.pcbi.1003482

**Published:** 2014-02-27

**Authors:** Thomas Garcia, Leonardo Gregory Brunnet, Silvia De Monte

**Affiliations:** 1 CNRS UMR 7625 Écologie et Évolution, École Normale Supérieure, Paris, France; 2 CNRS UMR 7625 Écologie et Évolution, Université Pierre et Marie Curie-Paris 6, Paris, France; 3 Instituto de Física, Universidade Federal do Rio Grande do Sul, Porto Alegre, Brazil; 4 CNRS UMR 8197 INSERM U1024, Institut de Biologie de l'École Normale Supérieure (IBENS), Paris, France; Pennsylvania State University, United States of America

## Abstract

The evolutionary stability of cooperative traits, that are beneficial to other individuals but costly to their carrier, is considered possible only through the establishment of a sufficient degree of assortment between cooperators. Chimeric microbial populations, characterized by simple interactions between unrelated individuals, restrain the applicability of standard mechanisms generating such assortment, in particular when cells disperse between successive reproductive events such as happens in Dicyostelids and Myxobacteria. In this paper, we address the evolutionary dynamics of a costly trait that enhances attachment to others as well as group cohesion. By modeling cells as self-propelled particles moving on a plane according to local interaction forces and undergoing cycles of aggregation, reproduction and dispersal, we show that blind differential adhesion provides a basis for assortment in the process of group formation. When reproductive performance depends on the social context of players, evolution by natural selection can lead to the success of the social trait, and to the concomitant emergence of sizeable groups. We point out the conditions on the microscopic properties of motion and interaction that make such evolutionary outcome possible, stressing that the advent of sociality by differential adhesion is restricted to specific ecological contexts. Moreover, we show that the aggregation process naturally implies the existence of non-aggregated particles, and highlight their crucial evolutionary role despite being largely neglected in theoretical models for the evolution of sociality.

## Author Summary

Although pervasive in the living world, collective behavior is a puzzle for evolutionary biology. The genetic traits that sustain it are costly for their carriers and make them vulnerable to the exploitation of asocial “free-riders” that benefit from the group without contributing to its cohesion. This paradox has spawned an extensive literature mainly concerned with elaborate cooperative behaviors that might be inoperant for simple biological entities such as microbes. We model successive life cycles of aggregation, reproduction and dispersal in a biological population combining a statistical physics approach to mimic the group formation process and an evolutionary game theory approach to account for the conflict between individual competition and collective success. Our results show a parsimonious way to the advent of sociality based on differential physical adhesion in organisms deprived of complex cognitive abilities. We also stress the key role of ungrouped individuals and specify the conditions on motion properties that make sociality possible. In detailing a mechanism akin to promote social behavior in microbes in the absence of genealogical relatedness, our work might shed light on both the maintenance of facultative multicellular lifestyles and the evolutionary origins of multicellularity.

## Introduction

The ability to form and sustain collective ventures is observed at all levels of the living world, spanning from human societies to microbial populations. In many biological settings, grouping requires individual traits that are costly for their carriers [1], [2], [3]. Sociality is thus an apparent paradox of evolutionary theory, as asocial “cheaters” who do not contribute to social welfare but reap group benefits should be favored by natural selection. This issue, that was first raised by Charles Darwin, has been revived in the last decades and is still pivotal in evolutionary biology, giving rise to an extensive literature on the evolution of cooperation.

The difficulty to explain the evolutionary emergence and maintenance of cooperation is particularly acute when the organisms diplaying social behavior are relatively simple such as microbes, so that they cannot rely on the complex cognitive and cultural abilities that are usually invoked as supporting cooperation in animals. Such simpler interactions and behaviors, together with the ease of cultivating bacterial populations in controlled environments, offer on the other hand the opportunity to address elementary mechanisms that underlie the evolution of sociality in general settings [1], [4], [5], [6].

Here, we consider the evolution of a social (adhesive and cooperative) trait in populations of organisms with a life cycle of aggregation-reproduction-dispersal, commonly observed in microbes displaying high degrees of cooperation, such as social amoebae or Myxobacteria [5]. In such cases, the existence of recurrent chimeric aggregates of potentially unrelated individuals appears to contrast with the classic expectation that cooperative behavior should be expressed to a lesser extent the weaker the genetical relatedness within social groups. We make the aggregation phase explicit by modeling cells as self-propelled particles moving on a plane, and we study under which conditions social traits evolve through natural selection, and to what spatial patterns they are associated.

Game theory has been long used to account for the evolutionary sustainability of genetically determined cooperative traits that benefit others while being costly to the individual [7]. Several different formalizations have been proposed that describe the effect of an individual's trait and its interactions on its own reproductive success. Among those, cooperation in social settings is classically modeled with 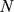-players games, where fitness depends on the features of the group. The simplest formulation of such games is the Public Goods Game (PGG) [8], where the benefits yielded by the group depend on the fraction of cooperators in it.

In questioning the mechanisms by which cooperative behavior can prosper, most models consider environments where group size is constant and independent of individual strategies. A growing number of studies has recently started to address cases when the interaction topology, and notably the size of the social groups, is an emergent property of individual-level features. These studies have modeled specific rules for entering groups with limited carrying capacity [9], [10]; evolvable preferences for group size [11]; competition for the use of a diffusible compound [12], or for empty space [13], [14]; the voluntary participation to groups [15], [16]; but also differential attachment supported by the cooperative trait [17], [18]. In many cases however, group formation has been modeled in well-mixed populations, or on a regular lattice where each individual occupies a cell and has a constant number of partners. While these assumptions are justified whenever individuals are either extremely motile or sessile respectively, they fall short in describing self-structuring traits in microbial populations with complex grouping patterns. More realistic models for the formation of groups from initially sparse individuals thus require an explicit account of particle movement in space and of the interaction forces that underpin the emergent “social landscape”.

With this respect, Self-Propelled Particles (SPP) models have proved useful to account for the formation of collective structures (e.g. swarms) based on simple local rules for interaction. Although SPP models have now become a primary tool to address collective behavior both in the physical and biological sciences, the exploration of their interplay with the evolutionary dynamics of individual traits is still in its infancy (but see [19], [20], [21], [22], [23]). In this work, we explore the conditions for a genetic costly trait that enhances interactions to evolve in a population, in a context where the aggregation scheme is explicit and the ecological and evolutionary timescales are separated. In the Methods, we define a SPP model in which particles exert interaction forces on their neighbors whose intensities differ according to their strategies, social (**S**) or asocial (**A**). After a fixed number of timesteps, individuals are assigned a fitness according to their strategy and the cohesiveness of their group. In the Results, we discuss the ecological dynamics of aggregation and the evolutionary trajectories of the social frequency across generations in this setting. Finally, we highlight the key role of microscopic parameters on the evolutionary dynamics, and stress that sociality might be promoted only in specific ecological contexts.

## Methods

In this section, we motivate and describe our model, which combines a Self Propelled Particles framework and a linear Public Goods Game. Cells are modeled as a population of particles differing in their adhesineveness that undergo successive cycles of aggregation, reproduction and dispersion, so that groups are “ephemeral”, and not persistent, structures [24]. Such description is not only relevant to understand the evolution of facultative multicellular microbes, it also provides a “thought experiment” to test hypotheses on the origins of multicellularity itself [25], [26], [27]. In the case when groups are persistent, the evolution of cooperation is made easier by mechanisms such as colony growth, low dispersal and incomplete separation after cell division [12], [28], [29], [30].

Within a cycle, the aggregation phase is ruled by a SPP, and is followed by a reproduction phase where particles leave offspring according to their payoff in a PGG. Particles are then dispersed, so that interactions in the following generation bear no memory of their previous positions. At the following generation, groups are thus formed again by genetically unrelated individuals. Iterating this cycle across many generations, we compute the evolutionary trajectory of the social trait.

### Aggregation model

We design a minimal model for collective motion that represents microbial populations with self-propelled particles moving on a plane. This kind of models has been widely explored in statistical physics (e.g. [31], [32], [33]), demonstrating that simple short-range interactions are sufficient to achieve spatial repartition of particles (typically of one single type) into clusters.

More in particular, it draws inspiration from Myxobacteria and Dictyostelids, that upon nutrient exhaustion abandon a solitary lifestyle to form multicellular aggregates. Even though interactions between cells are very complex, and several models have directly addressed specific features (e.g. the role of cyclic AMP oscillations and chemotaxis in the aggregation of *Dictyostelium discoideum* [34], or that of the rod shape in *Myxococcus xantus* streaming [35]), the present model retains only few essential characteristics of the aggregation process and investigates their evolutionary consequences.

In the absence of interaction, cells display a persistent random walk – i.e. with correlation between successive step directions [36] – as shown correct for the vegetative phase of Dictyostelium life cycle [37], [38], the directed and stochastic components being modeled here by force and noise terms respectively. The interaction forces may result from chemotaxis or adhesion proteins at the cell surface [39].

Natural microbial populations display differences in interaction modes (for instance, in stickiness or responsiveness to chemotaxis), that are often associated with differences in the capacity of specific strains to be overrepresented in spores [40]. Our modeled populations are composed of particles of two types –“social” (**S**) and “asocial” (**A**) – that have distinct interaction forces intensities, **S** particles being more attractive than **A** particles. The interaction strategy is deemed genetically encoded and unconditional. Previous models in which self-propelled particles differ in some microscopic feature (e.g. adhesion or motility) have been designed to study cell sorting within tissues [41], [42], [43], where the phase of aggregation from a dispersed initial condition was irrelevant.

**Figure 1:**
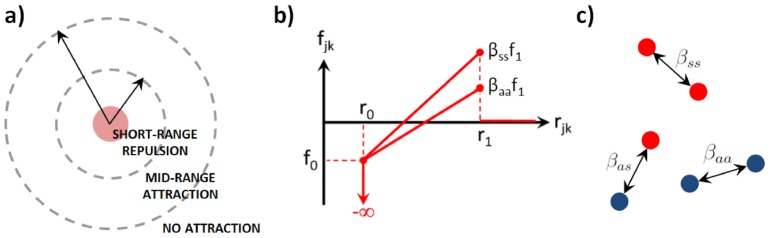
Local rules for interaction. Panel a: each individual undergoes an interaction force from its close neighbors (i.e. within a radius 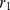); panel b: this force is repulsive within a radius 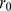 and becomes attractive until a radius 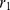; panel c: the interaction forces between two individuals are modulated by a coefficient depending on their respective strategies: 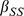 if both are social, 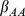 if both are asocial and 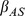 if one is social and the other asocial. We assume 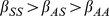.

We consider a population of 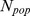 particles, either **S** or **A**, moving on a square of side 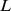 with periodic boundaries. Irrespective of the orientation of their velocity vector 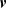, particles move all at the same speed 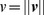, reflecting the inherent ability of propulsion of cells. Every particle has a finite spatial extension and exerts an interaction force on other particles as a function of their distances, as one would expect from physical adhesion and volume exclusion. Let us consider a particle indexed 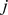 of type 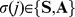. A second particle 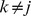 exerts a force 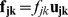 (where 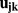 is a unitary vector directed from 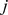 to 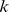) upon the focal particle 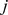. The dependence of the force 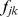 on the distance 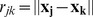 between particles 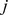 and 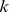 is illustrated in Figure 1. It is infinitely repulsive at short range (within a hard-core radius 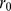 to account for the particle's spatial extension), null at long range (above the “interaction radius” 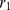) and is otherwise a linear elastic force:
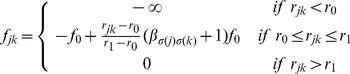
(1)This force reflects the existence of a finite action range of a glue, that keeps cells apart at an equilibrium distance around which they fluctuate below a cut-off radius. Cells may also interact via signaling, so that the interaction potential is continuous. We expect that as long as the interaction remains short-range, the model will be qualitatively unaffected, as occurs to the phase diagrams of SPP models where different kinds of forces have been tested [33], [41].

The coefficient 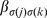 tunes the effect of the force exerted by 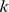 on the movement of 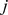, which depends on the particles' types. It thus can take four values 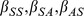 and 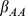. Consistently with the hypothesis of differential attachment, we assume that 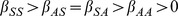. As a consequence, the equilibrium radius 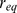 such that 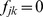 is shorter in **S**-**S** interactions than in **A**-**A** interactions (see Figure 1b). In the following, 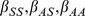 are in geometric progression (that is, 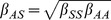) so that sociality entails a *differential* propensity to attach to other particles, but not a *preferential* bias toward other **S**s, when compared with asociality [18], [44]. This means that a focal **S** particle gets attracted to **S**s and **A**s in the same proportions as a focal **A** particle does, as 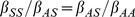; only to a larger amount. This warrants that no preferential assortment of strategies takes place just because of the choice of interaction intensities parameters.

For each particle, the direction of motion 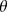 is updated according to the resulting force; at time 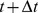, the velocity of particle 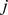 is 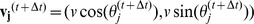, where
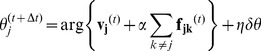
(2)with 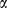 a coefficient with dimensions of a speed/force (in what follows, 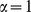) and 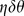 an additive noise randomly drawn between 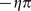 and 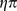 : 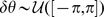. The position of each particle at time 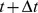 is computed accordingly:
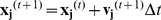
(3)The ecological dynamics resulting from this scheme of aggregation is detailed in the Results.

### Social dilemma

The aggregation process is stopped after a fixed number of timesteps 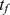, that defines the “ecological” timescale of the system. 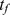 reflects the finite time before novel reproduction/death events. After aggregation, the population is segmented into groups according to a criterion described in the Text S1 of the Supplementary Information (SI). Such clustering algorithm allows us to attribute to each particle the size of the group it belongs to, as well as the number of **S** particles within this group. The definitions of these observables are detailed in Text S2 of the SI.

Once aggregation is over, the reproductive success of every particle is determined as its payoff in a PGG played within its group. The PGG is a simple form of social game, that gives rise to the so-called tragedy of the commons [45]: individuals who contribute to the public good are disadvantaged with regard to non-contributing co-members, even though gains are maximal when everybody takes part to the common endeavor. The common good at stake here is group cohesion itself, so that the payoff individuals derive from their group depends on the proportion of **S** members [18]. Sociality thus plays a role both in the aggregation process and in the performance of groups. This assumption is consistent with what happens in several social microorganisms, where cell adhesion is a major factor determining the cohesiveness (and as a consequence, the viability) of cell aggregates [46], [47], [48]. Equivalent assumptions have also been made in the theoretical literature e.g. in [49]: there, adhesive social cells entail higher group sizes and large groups are supposed more viable than small ones, leading to the maintenance of sticky cells through the interplay of the individual and the group levels.

In the simplest form of a PGG, each **S** contributes 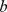 to group cohesion at a cost 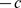 to its reproductive success, whereas **A**s neither contribute nor pay a cost. Within a group, contributions are summed and shared among all members, irrespective of their strategy. Benefits thus scale linearly with the proportion of **S**s in a group. Other formulations of PGGs account for non-linear dependencies of the benefits on the number of contributors [50], [51]. In order to disentangle the non-linear effects of group formation from those introduced by more complex forms of the game, we restrain here to the linear case. We will however briefly discuss the expected effects of different PGGs in the Results.

In line with [52], we can separate an individual's payoff in a payoff due to self (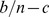 for **S**s, as they get a share 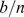 of their own contribution and pay a cost 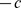, and 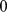 for **A**s) and a payoff due to the group co-members (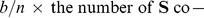 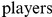). Singletons do not earn any group-related benefit, and thus have a payoff of 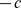 or 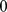 depending on their type. If a focal individual belongs to a group of size 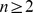, 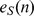 and 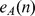 respectively denote the average number of **S** co-players in its group, conditional to its own strategy **S** or **A**. The average payoffs of an **S** and an **A** particle in groups of size 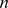 are thus:
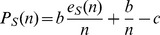


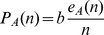



If the social trait has no effect on the groups an individual belongs to (as for instance for randomly formed groups of fixed size 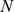), there is no positive assortment between **S**s and 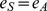: sociality outcompetes asociality only when 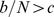, that is when a **S** individual's share of its own contribution suffices to make its investment profitable (*direct benefits* case). Excluding this trivial case, sociality provides an average advantage within groups of fixed size only if sufficient assortment within strategies (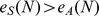) tips the balance in its favor (*altruism* case). In more general cases, when a group formation process produces groups of different sizes and different compositions, the social trait assumes a different status (directly beneficial or altruist) depending on the realized population structure, and may change along an evolutionary trajectory.

### Evolutionary algorithm

The evolutionary trajectory of the population throughout successive generations is obtained by numerically evaluating the payoff of every particle at the end of the aggregation phase. At any given generation, the mean payoffs of **S**s and **A**s, which depend on the population structure at time 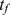, provide their respective reproduction rates (see Text S3 of the SI for details). Particles then may die, irrespective of their strategy, with a density-dependent mortality rate that keeps the total population size close to a fixed carrying capacity.

At the end of the generation, the resulting population is dispersed: the position and orientation of each particle is randomly assigned at the beginning of the next generation's aggregation phase. The complete re-shuffling of particles corresponds to the worst-case scenario in which assortment at one generation cannot be maintained (and enhanced) in evolutionary time. Sociality would be further favored if the spatial structure was inherited, so that cooperative traits may be boosted by groups engaging in between-group competition [53], [54].

The probability to leave offspring is obtained normalizing the particle's payoff in a range 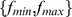. For weak selection strengths (that is, small 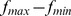), the evolutionary trajectory generated by this algorithm is well approximated by a continuous-time replicator equation. According to this equation, the only determinant of the variation in **S** frequency is its average payoff advantage with respect to the **A** strategy. In the Results, we show that such average payoff can be expressed in terms that reflect different features of the population structure at the end of the aggregation phase.

In the simulations, the evolutionary algorithm is iterated for a number of generations (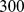) sufficient for the frequency in the population to reach a stable equilibrium state. The algorithm is described with more details in Text S3 of the SI.

## Results

Precising the process by which particles interact and form groups allows us to study the interplay of the ecological timescale – relative to the aggregation phase – and the evolutionary timescale, over which the frequencies of social and asocial strategies change in the population. In the following sections, we will examine these timescales separately and eventually discuss what are the features at the particle level that support the evolution of sociality, and to what population structures this evolution is associated. First, we focus on the outcome of the aggregation step within one generation. Then, we address the evolutionary dynamics of the social trait across generations and highlights the role of *assortment* and *volatility* in determining the success of the social strategy. Finally, we describe the dependence of the evolutionary equilibrium on microscopic parameters of motion and interaction.

### Local differences in adhesion govern group formation and spatial assortment in the aggregation phase

Within one generation, particles interact for a finite number of timesteps 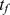, according to the numerical model described in the Methods. Initialized in random positions, particles will aggregate or not in groups depending on the ecological parameters, analogously with what is observed for other models of SPP [31], [32], [33]. Our simulations being halted before the asymptotic state is reached, we are focusing on the clustering of the population into groups that occurs on a fast timescale, and we neglect all features such as group diffusion, merging and internal reassortment that occur on longer timescales. Slow relaxation to the asymptotic state mainly induces sorting within groups, which in our model has no fitness effect, hence no qualitative effect on the evolutionary outcomes.

The population forms groups in a broad range of the parameter space, but the size distribution of the groups and the proportions of grouped vs. free particles depend on the ecological parameters ruling particle motion and interaction. We refer to the online Supplementary material for movies showing simulations of the process of group formation. These movies bear a strong resemblance with low magnification movies of the aggregation of *D. discoideum*, where initially dispersed cells form clumps of different sizes, while some cells keep moving outside the aggregates.

**Figure 2:**
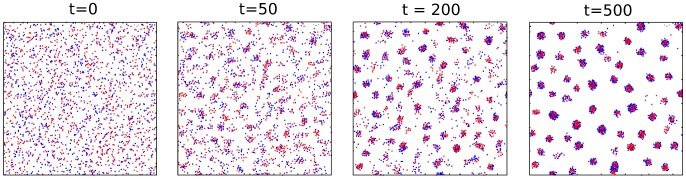
Simulation of a population undergoing an aggregation process. The group formation process is such as described in the Methods, here displayed at different timesteps. **S** particles (red) are more adhesive than **A** particles (blue). At each timestep, every individual updates its position and direction according to its neighbors within a radius 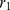. Individuals begin to form clusters and then to spatially re-arrange themselves within clusters. Some of them may remain alone (not attached to any group). At the end of the aggregation phase, a spatial criterion enables to automatically clusterize particles into groups groups and to determine each group's size and composition. Parameters: 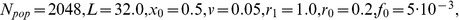 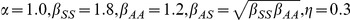.

The population remains scattered and no group is recognizable when directed motion overcomes the interaction forces. This occurs when the interaction cut-off radius 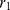 is short; the population density 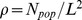 is small; the noise level 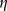 is high; the velocity 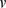 is large. Otherwise, local fluctuations are amplified and particles start to cluster until a quasi-steady state is reached where most particles are clumped into groups of different sizes and densities, while some particles move in the “gas” phase between groups (Figure 2). Within groups, each particle vibrates around an equilibrium position corresponding to the balance between all attractive and repulsive forces exerted by its neighbors, plus the noise component. Groups typically have a circular shape and are separated by a distance of the same order of magnitude as 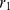.

In many parameters regions, and as long as 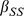 and 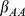 are different enough, groups display spatial segregation with more adhesive particles occupying equilibrium locations at the group core and less adhesive particles gravitating at the periphery in a unstable fashion. Such sorting within aggregates of cells with heterogenous adhesion, motility or chemotactic properties is a well-studied phenomenon [41], [42], [43], [55], [56], [57]. It resembles moreover the observed spatial segregation between WT flocculating cells and non-flocculating cells in species like *S. cerevisiae* [47]. In more *ad hoc* models, the influence of within-group structure on particle fitness might be implemented. For instance, we expect the evolution of sociality to be further favored if the public good is explicitly modeled as a diffusible substance [58], [59], thus reducing further the potential benefits of cheater cells that are found at the periphery of groups [60].

### Assortment and differential volatility between strategies drive the evolution of sociality

On the evolutionary timescale, the fate of the social type hinges upon the emergent structure of the population after the aggregation process. In our simulations of the evolutionary dynamics, the population always stabilizes to a monomorphic equilibrium, either fully social or fully asocial. This is probably the consequence of the simple linear form of the PGG, which generally does not support the coexistence of different strategies. The population structure that is achieved at the evolutionary equilibrium depends on the microscopic features of the dominant particle type, and falls under three categories: asocial and grouped; asocial and dispersed; social and grouped. A fully social dispersed equilibrium is always evolutionarily unstable: indeed, if **S** particles are unable to form clusters, they do not get any group benefits and are thus defeated by **A** particles that do not pay the cost of sociality. In the case of a fully asocial equilibrium, the population can either remain dispersed or grouped, depending on the ecological parameters.

**Figure 3:**
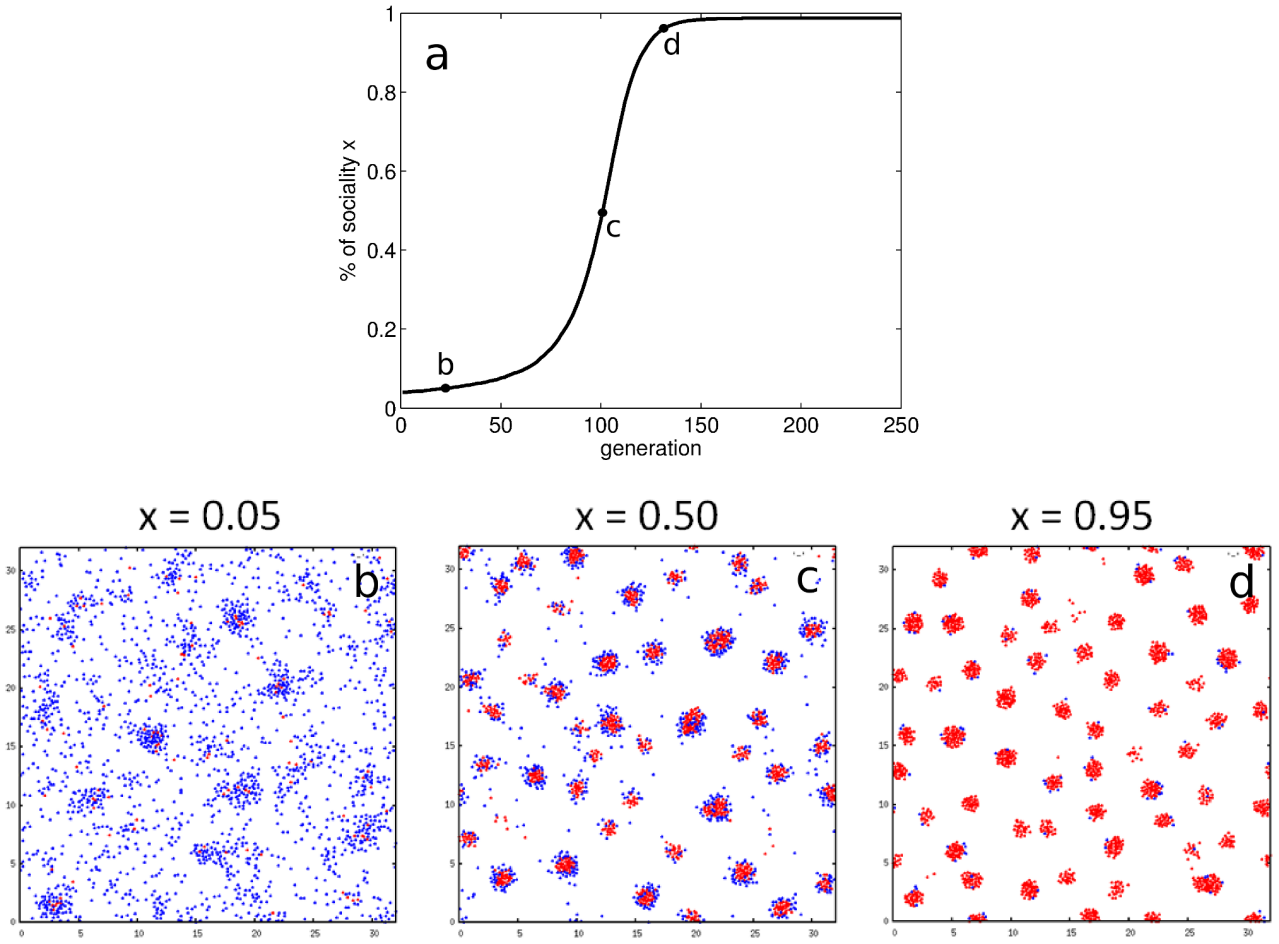
Evolutionary dynamics of the social trait and population structure. Panel a: **S** mutants, initially in small frequency, are favored as they derive more net benefits from groups on average than **A** particles, and ultimately invade the population. Parameters: 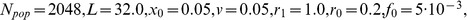 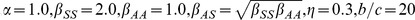. Panels b, c, d: snapshots of the population after the aggregation step (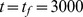) at frequencies 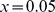, 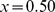 and 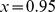 during the evolutionary trajectory depicted in panel a. As 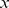 increases, the population gets more and more clustered and free individuals fewer and fewer. When **S**s and **A**s coexist in the population, groups tend to be spatially segregated with **S**s strongly bound at their core and **A**s loosely attached at their periphery. As a consequence, more **S** individuals find themselves grouped and **S**s tend to be better off than **A**s.

Figure 3 recapitulates the evolutionary dynamics observed in the cases when sociality takes over the population. It displays the frequency 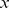 of **S**s in the population through generations (Figure 3a), starting from the initial condition 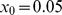, and the spatial pattern achieved at the end of the aggregation phase (the ecological timescale is hidden here). **S**s initially have a higher average payoff than **A**s and thus replicate faster. The evolutionary feedback on the ecological timescale thus boosts **S** particles, that in turn give rise to larger groups, and ultimately leads to the fixation of sociality in the population. When **S**s are rare (Figure 3b), group cohesion is low and most of the particles remain ungrouped. When 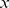 increases (Figure 3c), groups are nucleated by a hard core of **S** individuals. **A**s thus get less benefits from groups. Finally, when sociality has invaded the population (Figure 3d), groups are much more cohesive and very few individuals are ungrouped. Mean group size saturates, and groups become denser since equilibrium distances are shorter among socials.

The condition for **S**s to be favored over **A**s depends on the game parameters 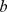 and 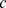, as well as on the emergent population structure shaped by the ecological parameters (form and intensity of the forces, population size and density, noise level, velocity, radii of interactions) that govern the relative importance of diffusion (the persistent random motion) vs. cohesion (the interaction forces). If non-linear payoff functions are chosen, the evolutionary success of sociality may as well depend on other game parameters, e.g. a threshold to activate the public goods [50] or a synergy/discounting rate [51]. Once the population structure is known, however, such condition can be expressed in terms of the evolutionary parameters and of two aggregated observables that quantify statistically the effect of population structure on particle assortment.

Following [18], we can derive a simple condition for sociality to be favored in a population after aggregation. Let us define 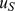 (resp. 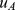) the proportion of **S** (resp. **A**) particles that remain *ungrouped* at the end of the aggregation step, and 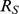 (resp. 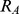) the average fraction of **S**s experienced in the group of a focal **S** (resp. **A**) particle. According to the notations of the Methods section, 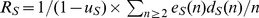 and 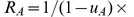 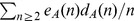, where 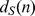 and 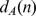 are the probability distributions for a **S** (resp. **A**) particle to be in a group of size 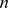. The terms 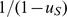 and 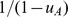 appear as these values are calculated conditioned to the fact that the **S** or **A** particle is not a singleton. The condition for the social trait to be favored at this generation is:
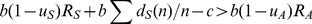
(4)


The 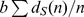 term in the LHS corresponds to the average marginal gain an **S** particle gets from its own contribution in the PGG within its group. The frequency of sociality therefore increases at the next generation as soon as the aggregation process entails sufficient differences in *assortment* (i.e. 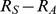 is large enough) or *volatility* (i.e. 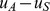 is large enough) between **S**s and **A**s.

Such two macroscopic quantities can be in principle measured experimentally in microbial populations by mixing two strains stained with different fluorescent markers. The main obstacle to quantify them precisely is a technical one: such measure requires to resolve single cells and at the same time to span a sufficiently wide field so that many aggregates are visible.

### Parameters of motion and interaction condition the evolution of sociality

Sociality can get established as an effect of the feedback between ecological processes – linked to the emergence of particle aggregates – and the evolutionary change in frequencies of each type: it is favored when the microscopic aggregation parameters create a sufficient degree of assortment within groups and enhance group volatility. We explore now when these conditions are met as a function of four fundamental parameters underpinning particle motion and interaction: noise intensity, particle velocity, interaction radius and population density. The effects of changes in particle diameter 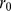 can be also understood based on this analysis, since its value is directly obtained by rescaling the other parameters. Although in some cases random mutations and finite-size fluctuations are sufficient to cause the evolutionary invasion of social particles, most often the initial fraction 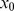 of **S**s must exceed a threshold in order for the positive eco-evolutionary feedback to get established. Therefore, we initialized the system with 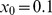. The diagrams in this section illustrate how qualitatively different regimes can be attained as the microscopic parameters are changed, and how two different kinds of transitions between them are understandable with regard to the emergent population structure.

#### Noise intensity

**Figure 4:**
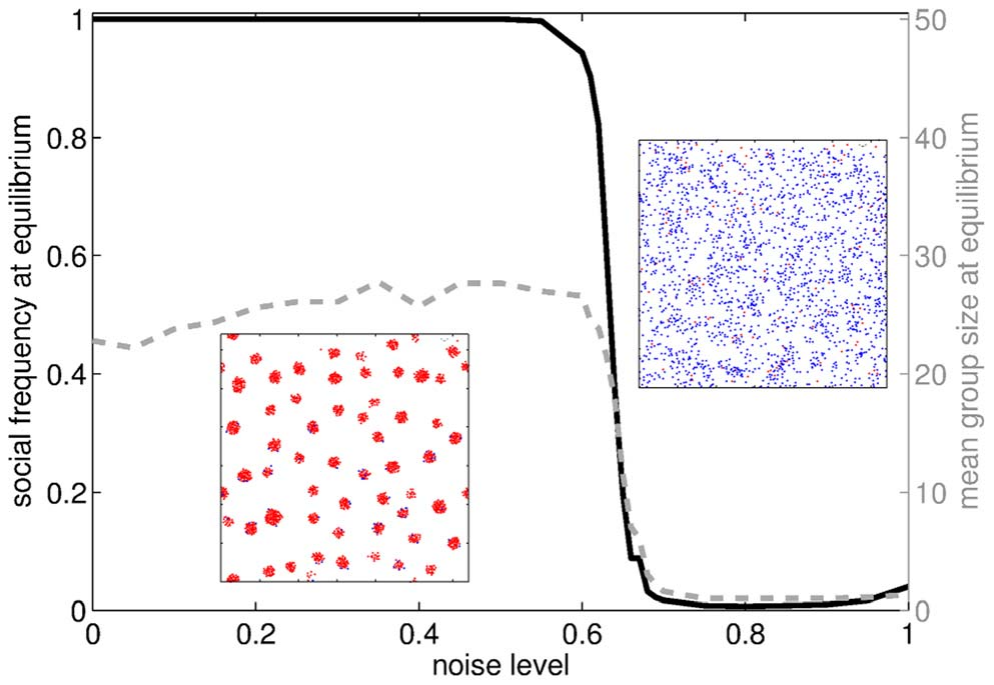
Evolutionary endpoints and mean group size at equilibrium when the noise parameter 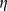 varies. Two phases can be observed: for low values of 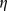, particles form groups and **S** players are favored until invasion of the population; for values of 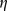 exceeding a threshold, individuals are not able to aggregate anymore and sociality is no longer profitable: the final state is a dispersed, asocial population. Insets: snapshots of the population after the aggregation step (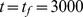) at the evolutionary equilibrium. The two snapshots are representative of the low and high noise regimes, respectively. Parameters: 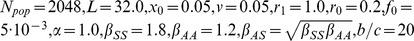.

The noise parameter 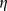 quantifies the extent to which random perturbations override the interaction forces between particles. A value of 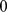 for 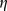 corresponds to the case when a particle's direction at each timestep is completely determined by its current direction and the total force exerted by its neighbors. Conversely, a value of 1 means that its direction is chosen uniformly randomly within the range 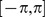, so that the particle undergoes uncorrelated Brownian motion. A sharp transition is observed between a regime of clustered, highly social populations when noise is low and a regime where the population remains dispersed and asocial when the noise exceeds a threshold value (Figure 4). In the first phase, noise is low enough to keep **S** particles together once they have joined a group, as they are bound by strong interactions forces. On the same timescale, instead, **A** particles, that are linked by weaker interactions, aggregate less (thus collecting less often group benefits than **S**s), and experience less social group environments when they do. Above the noise threshold, particles of any type become detached from each other and the population is highly volatile, as reflected by the concomitant drop in group sizes.

A certain level of inertia is thus required for a social variant to be selected in a population. This suggests that turbulent environments might be less favorable to the establishment of social behavior also because they hinder the formation of groups, other than because they offer a smaller number of niches to drive the evolution of more adapted types [61].

#### Particle velocity

**Figure 5:**
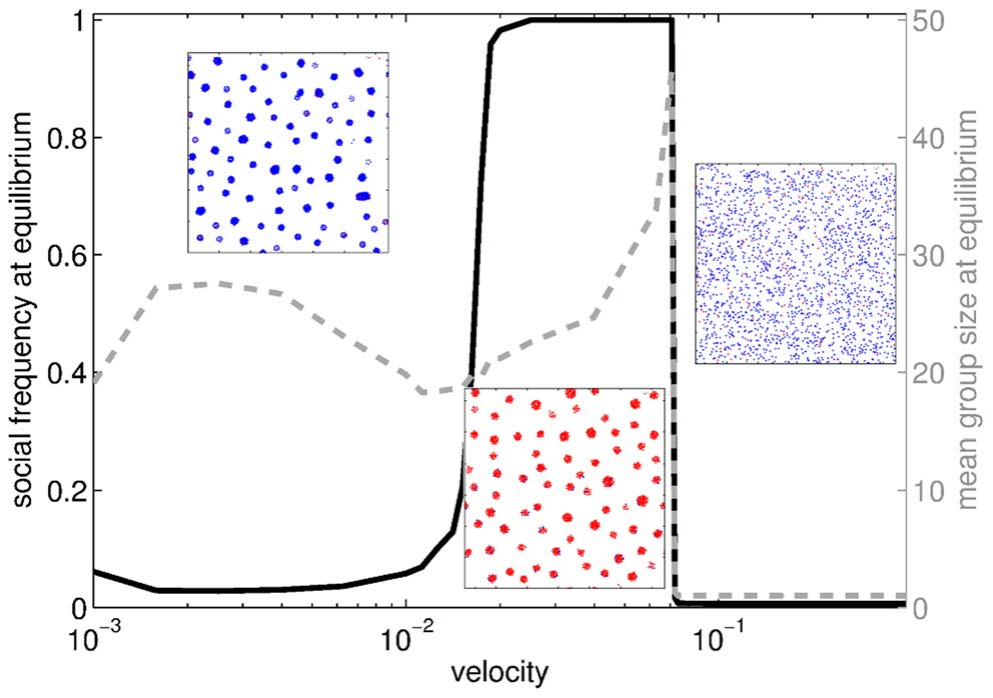
Evolutionary endpoints and mean group size at equilibrium when the velocity 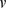 varies. Three phases can be observed: for small 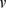, particles form groups that are poorly assorted between **S**s and **A**s, so that sociality is not profitable enough to offset its cost: **A** players dominate at the evolutionary equilibrium; for intermediate 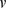, groups are more volatile and sufficient assortment between **S**s occurs to promote sociality: **S** players dominate at the evolutionary equilibrium; for high 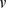, individuals are not able to aggregate anymore and sociality is no longer profitable: the final state is a dispersed, asocial population. Insets: snapshots of the population after the aggregation step (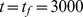) at the evolutionary equilibrium. The three snapshots are representative of each regime. Parameters: 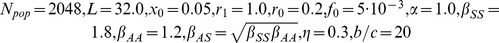.

The same effect observed for high values of noise also occurs for high velocities, that make interaction forces insufficient to hinder volatility. Figure 5 displays the same kind of transition from dispersed, asocial evolutionary equilibria to grouped, social populations as the velocity decreases. However, a transition of different nature can be seen when the velocity is diminished further. Groups keep forming when particles are slow, but their composition is mostly determined by the positions of particles before aggregation, and is thus close to random assortment. Contrary to the transition occurring at higher velocity, groups keep existing across the transition, and their size does not drastically vary. The intermediate interval where sociality thrives corresponds to speeds that are sufficient to hinder cohesion among **A**, but not **S** particles.

The conflict between diffusion and cohesion (i.e., between speed of movement and interaction forces) thus results in a range of velocities where **S** particles are assorted and poorly volatile, while **A** particles are strongly volatile. Elsewhere, either **S**s are too poorly assorted, or the population is too volatile. This suggests that the environments that promote social adhesion are those that are neither too fluid nor too viscous. These results are consistent with other recent studies: Meloni and co-workers [62] discuss a model in which agents move freely with constant speed on a 2D-plane (thus with no interaction forces between them) and play a prisoner's dilemma game with their closest neighbors at each timestep. They found that high velocities are detrimental to the evolution of cooperation, as the neighborhood of each particle then resembles a well-mixed population. The effect of velocity on the evolutionary dynamics is even closer to our model when the game is changed to a PGG, showing a similar rise-and-fall pattern [63].

#### Interaction radius

**Figure 6:**
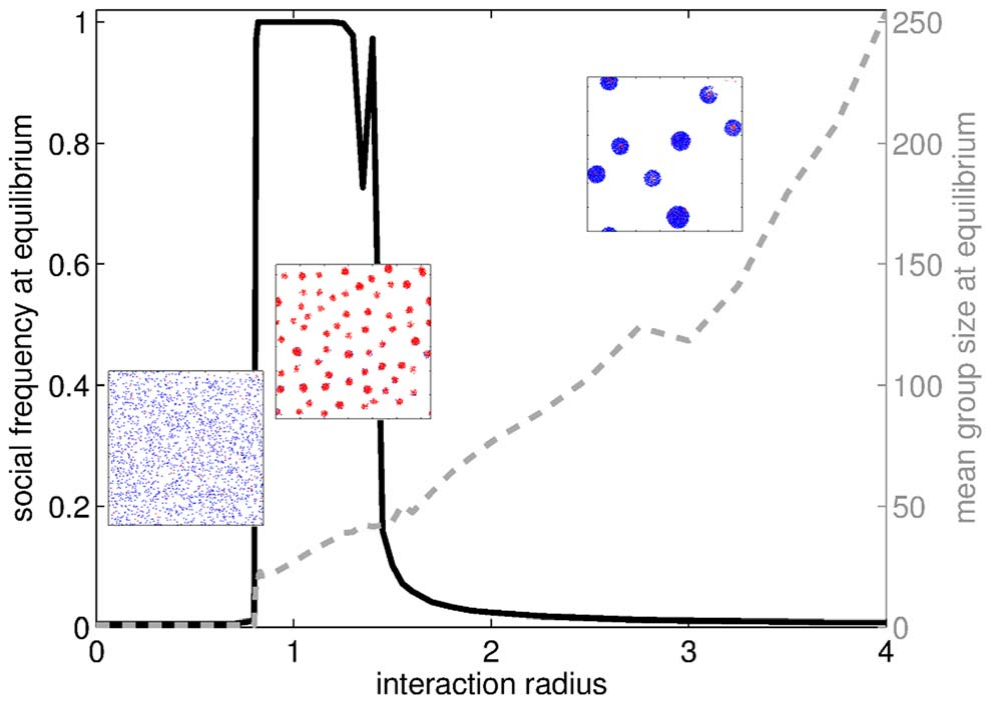
Evolutionary endpoints and mean group size at equilibrium when the interaction radius 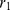 varies. Three phases can be observed: for small 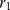, particles do not manage to form any group and asociality takes over the population; for intermediate 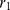, groups form such that differential volatility+assortment of **S**s combined favor sociality until invasion; for high 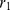, interactions resemble that in well-mixed populations (so that assortment between **S**s is low) and very few individuals remain ungrouped (so that differential volatility is low), thus impeding the advent of sociality. Insets: snapshots of the population after the aggregation step (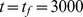) at the evolutionary equilibrium. The three snapshots are representative of each regime. Parameters: 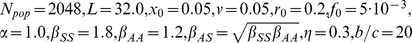.

The same two transitions are observed when the interaction radius is changed (Figure 6). When 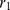 is low, particles are not able to form clusters: each particle's neighborhood is too small for cohesion to overcome diffusion and the population remains in the gas state. The transition to a fully social evolutionary equilibrium is concomitant with the appearance of groups. When 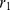 increases, particles experience more populated neighborhoods and the resulting forces exerted on them are sufficient to compensate for **S** particles', but not **A** particles', diffusion. For still higher interaction radii, particles experience large, close to well-mixed neighborhoods, and volatility remains low, so that assortment is closer to random. In the end, sociality is no longer profitable and vanishes. Whatever the winning strategy, the average group size in the population keeps increasing as the interaction radius becomes larger.

It appears that both short-range and long-range interactions are detrimental to the advent of sociality. Similarly to the case of the velocity, the interaction radius must belong to an intermediate range so that the **S** strategy is sufficiently assorted to be selectively advantaged.

#### Density

**Figure 7:**
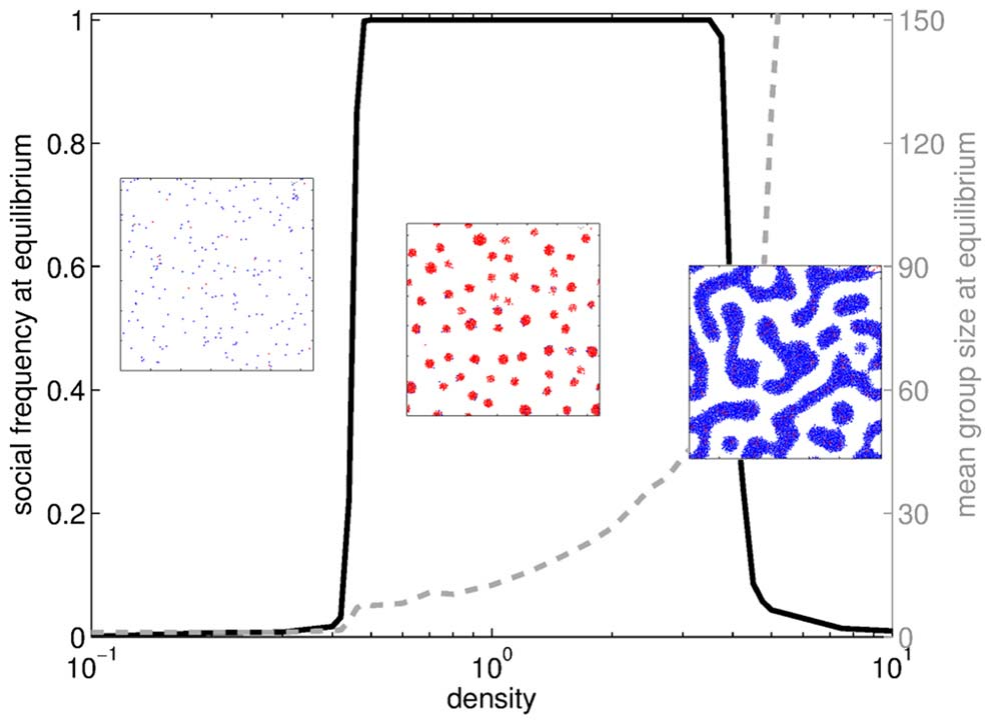
Evolutionary endpoints and mean group size at equilibrium when the density 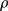 varies. Three phases can be observed: for small 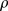, particles are too dispersed for interaction forces to overcome directed motions so that no group forms and asociality dominates; for intermediate 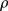, groups form such that differential volatility+assortment of **S**s combined favor sociality until invasion; for high 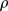, interactions resemble that in well-mixed populations (so that assortment between **S**s is low) and very few individuals remain ungrouped (so that differential volatility is low), thus impeding the advent of sociality. Insets: snapshots of the population after the aggregation step (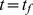) at the evolutionary equilibrium. The three snapshots are representative of each regime. Parameters: 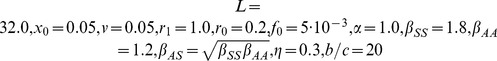.

Here again, we observe a rise-and-fall pattern of the social frequency at equilibrium as a function of the population density 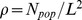 (Figure 7). When density is too low, particles are too distant to form clusters within time 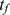, so that **A** particles are favored and take over the population. When density is too high, particles are close to one another and very few of them are left alone, decreasing the effect of differential volatility, hence favoring **A** particles anew. Sociality can only invade the population when density is restricted to an intermediate range. Outside this range, either the absence of groups or the intensity of the competition favors the less costly type. A similar result has been found in [62] with a different model for individual motion and social game.

## Discussion

The evolution of social behavior is a riddle for evolutionary biology because of the disruptive power of within-group competition between individuals that invest or not in the public good. In microbes and in the early stages of the transition to multicellularity, most mechanisms that sustain coperation through the use of cognitive abilities are ruled out. On the other hand, genetic relatedness may not be the only driver of the evolution of collective behavior, since microbial aggregates are commonly observed even when cells of potentially different origins come together. Here, we have explored one possible mechanism that allows the evolution of sociality and sizeable groups, when sticky self-propelled particles moving on a plane are assigned a fitness that depends on their social environment. The emergent structure of the population, underpinned by the adhesion forces between particles, feeds back onto the evolutionary dynamics of more or less adhesive types. By analyzing a model that comprises successive cycles of aggregation-reproduction-dispersal, we have shown that sociality gets established in a limited range of parameter values: intermediate particle velocities; intermediate interaction radii; sufficient persistence in the particle directed movement; intermediate densities. These results can be understood in terms of two features of the population structure: assortment within groups and volatility, both of which affect the average fitness of particles with different interaction forces.

Assortment among types has long been pointed out as a requirement for the evolution of costly cooperative behaviors (e.g. [52], [64], [65]). However, experimentalists as well as theorists still debate on how such assortment is actually reached when genealogic relatedness does not appear to play a central role. Most solutions involve some kind of recognition of other individuals' strategies, or at least information about a variable correlated with the strategy: a “green beard” gene coding for both the character, the recognition of copies of the genes among partners and preferential interaction with their carriers [66]; partner choice [67], [68]; conditional strategies involving choosiness [69]; matching by group size preference [11]; direct [70], [71] or indirect/generalized reciprocation [72], [73], [74], etc. Here, we describe a mechanistic process by which particles are endowed with attractive forces that are independent of the social context. Assortment occurs with no need to assume that **S** particles attach *preferentially* with other **S**s; only *differential* attachment of **S**s and **A**s is required. This important distinction has been alluded to or stressed in several recent works [18], [47], [75]. Ultimately, **S** particles enjoy the advantages of group cohesion to a larger extent, to the point that they can offset the cost of sociality.

Volatility is a much more neglected factor to achieve distinct reproductive successes for each type. Differential volatility means that asocials are less prone to be in a group, or at least more loosely stuck to the group; therefore, more likely to get no group benefits or a lesser share of them. Surprisingly, the possibility that individuals do not participate in any group has been overlooked in models of the evolution of cooperation. In the papers that did, being alone results either from an encoded strategy (e.g. [15], [16]) or to coercion by cooperators [76], rather than being a by-product of an explicit group formation process. Recently, detailed models of motion began to be implemented in evolutionary models that allow in principle for the existence of lonely individuals [62], [63]. Indeed, in many actual group-structured biological populations, a proportion of individuals typically fail to join any group [47]. We stress that, as soon as the proportion of ungrouped particles differ for both types, the evolutionary dynamics is affected in favor of the more strongly aggregating type. In our model, differential volatility occurs as the cooperative trait is related to grouping ability itself, as stronger adhesion forces confer cohesiveness to groups but also enhance individual attachment. Any cooperative trait increasing the probability to end up in a group would yield qualitatively similar results: socials and asocials may be defined on the basis of differences in properties other than attachment, e.g. their interaction radius.

In general, assortment and volatility are not independent features of the emergent population structure. In our simulations, the faculty for **S** particles to become positively assorted comes along with a lesser tendency to be left alone by the aggregation process. However, it is noteworthy that, in situations when sociality is the winning strategy, positive assortment alone may not be sufficient to account for its advantage. Indeed, assuming that 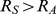 and 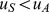, the two conditions 1) 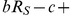 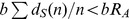 (assortment alone is not enough to favor sociality) and 2) 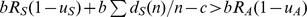 (assortment+differential volatility combined favor sociality) are compatible as soon as 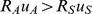. In this case, differential volatility drives the rise in frequency of sociality, while it would not be the case discounting singletons. This suggests that in real settings where group size is distributed and not fixed, models and experiments might overestimate the constraints for cooperative behavior to be favored. While positive assortment and differential volatility are two complementary effects that promote sociality, they both stem from the biologically plausible hypothesis that a character may affect the expected group size distribution. Examples of traits regulating group size are known in *D. discoideum* [38], [77], although their effects on the group size distribution have to our knowledge never been quantified.

We highlighted that parameters related to particle motion are key in the evolutionary success of social individuals. Noise, velocity, density and interaction radius must be restricted to specific ranges for sociality to be able to take over the population; otherwise asociality dominates. In actual biological settings, these parameters might have co-evolved with adhesion properties, and their evolutionary dynamics may be explored with a multi-trait model.

With our model, we show that observing and quantifying the properties of the population structure generated by a given mixture of strains may inform on the mechanisms that underlie the evolutionary process. The issue of being able to count a large amount of aggregates (so that statistics are reliable) can be overcome by means of microscopes screening a large surface and still maintaining a single-cell resolution [78]. Our analysis indicates what are the patterns that one would expect if the evolutionary experiments were carried out under different environmental conditions that affect cell-level properties, such as cell density or substrate hardness (that condition cell movement). More importantly, they indicate two statistics that may predict if a given population would evolve towards more or less sociality.

Although unicellular organisms are often found in large aggregates, their high dispersal abilities and the consequent mixing of genotypes makes the establishment and maintenance of social behavior apparently paradoxical. When physical mechanisms underlying the formation of groups are made explicit, however, the evolution of sociality looks less mysterious, and one can start asking quantitative questions on the processes that led to the emergence of cellular aggregates. The simple model presented here can be enriched with further details implementing additional features of microbial organisms, such as alignment terms [31], [33], an explicit account of the cell shape [35] and chemotaxis [79], [80]. The exploration of the mechanistic role of cell-cell interaction in shaping the social structure is a fundamental step to understand altruism in microbes, as well as the possible evolutionary paths towards multicellularity.

## Supporting Information

Text S1 **Description of the criterion used to determine groups.**
(PDF)

Text S2 **Definition of the observables.** We define the following observables related to the population structure, as they are relevant for the analysis of the evolutionary dynamics of the social trait: 1) the group size experienced by an average individual of each strategy; 2) the social ratio experienced by an average individual of each strategy; 3) the volatility of each strategy.
(PDF)

Text S3 **Details of the evolutionary algorithm used to perform the simulations.**
(PDF)

Video S1
**Animated GIF displaying an aggregation process.** The following parameters are used: 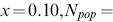 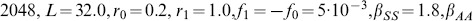 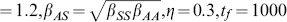. Here the population is mostly asocial and no distinct group forms during the aggregation step.(GIF)

Video S2
**Animated GIF displaying an aggregation process.** Same parameters as in video S1, except 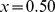. Here stable groups form during the aggregation step, that are nucleated by a hard core of **S** particles with **A** particles at their periphery. A proportion of particles remain alone (not bounded to a group), among those a larger part are asocial.(GIF)

Video S3
**Animated GIF displaying an aggregation process.** Same parameters as in video S1, except 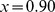. Here again, stable groups form during the aggregation step, that are on average larger and denser than when 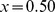. Very few particles remain ungrouped.(GIF)
