# Supplementary material for: Differential Adhesion between Moving Particles as a Mechanism for the Evolution of Social Groups
Source: PLoS Comput Biol. 2014 Feb 27;10(2):e1003482. doi: 10.1371/journal.pcbi.1003482 (PMC3937110; doi:10.1371/journal.pcbi.1003482)
Supplement: Text S1 — Description of the criterion used to determine groups. (PDF) [file pcbi.1003482.s001.pdf]

Text S1: Description of the criterion used to determine groups

In order to calculate statistics on aggregation as well as individuals' payoffs, we need to find a robust way to determine groups from the positions and movements of the particles in the last timesteps. A first step is to decide, for each of them, whether it is stuck within a group or not. A simple step-by-step procedure will then be applied on the individuals found to be in a group to clusterize the population. The results presented in the main text correspond to the following implemented criterion:

- assess the influence of the resulting forces that exert on an individual j over the update of its movement. The idea is that an individual in a group is under the influence of more attraction forces than a free individual. We then assess to what extent such forces affect the speed vector of an individual and we do it for n_{step} timesteps in order to average out fluctuations localized in time. A free individual is one that does not undergo sufficient attraction forces enough times during the last n_{step} timesteps:

$$j \text{ is grouped } \Leftrightarrow \sum_{t=t_f-n_{step}}^{t_f} \mathbb{1}_{\left\{||\sum_k \mathbf{f_{jk}}^{(t)}||/v>1\right\}} > \gamma\, n_{step} \tag{1}$$

  where γ is some threshold coefficient between 0 and 1 (e.g. 0.5).

This criterion focuses on the *a priori* causes of aggregation, and allows for a more or less stringent definition of "grouped", by tuning the parameter γ. The result of a segmentation of the population into groups according to this criterion is shown in Fig. S1. To check robustness, we also tested a second criterion that focuses on the effect of aggregation behaviour on individuals and their movement *a posteriori*:

- compare the distance covered by the focal individual j with that expected from a random walker. Indeed, for low to moderate noise values a grouped individual's movement is characterized by a shorter correlation length than with Brownian movement. We thus sample the positions of the individual j at n_{step} distinct, non-successive timesteps before the ending of aggregation, i.e. at times $\tau_0 = t_f - (n_{step}-1)l_{step}$, $\tau_1 = t_f - (n_{step}-2)l_{step}$, $\ldots \tau_{n_{step}-1} = t_f$, where l_{step} is the duration between two sampling events. If the cumulated distance covered by individual j if greater than the average cumulated distance covered by a random walker ($= v \cdot n_{step}\sqrt{l_{step}}$), it is considered as free, otherwise it is deemed grouped:

$$j \text{ is grouped } \Leftrightarrow \sum_{k=0}^{n_{step}-1} ||\mathbf{x_j}^{(\tau_{k+1})} - \mathbf{x_j}^{(\tau_k)}|| < v \cdot n_{step}\sqrt{l_{step}} \tag{2}$$

We found that this second criterion leads to comparable separations between grouped and non grouped individuals in most cases; for very large noise values (such that the interaction forces and current speed vectors play little role in the update of movement compared to the random component), it however inaccurately deems some individuals

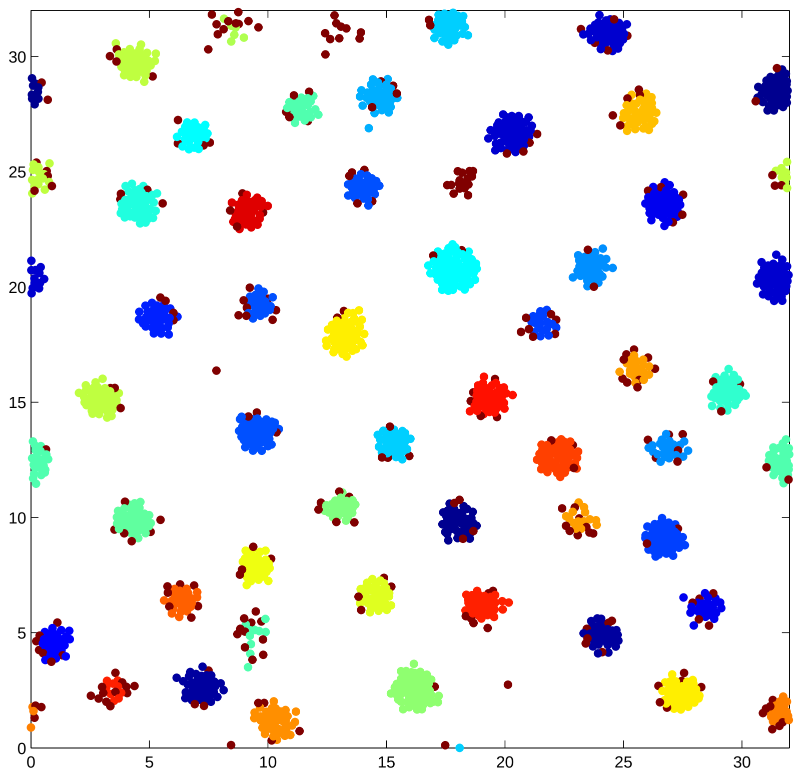

Figure S1: Application of criterion 1 on a population after the aggregation step. In this picture, each group determined by criterion 1 is assigned a random color and individuals that are deemed free are represented in dark brown. Some of them are isolated while others are found at the periphery of groups or within small, unstable groups.

grouped whereas none is, as correlation lengths are now all comparable to that of a random walker.

Once we decided which individuals are free and which are in a group, the actual groups are determined applying a simple recursive procedure on grouped individuals: any grouped individual with a radius r_1 of another grouped individual belong to the same group. In the end, for each individual j we are able to compute the size n_j of the group it belongs to as well as, if $n_j > 1$, the number s_j of social individuals in its group.
