# Supplementary material for: Differential Adhesion between Moving Particles as a Mechanism for the Evolution of Social Groups
Source: PLoS Comput Biol. 2014 Feb 27;10(2):e1003482. doi: 10.1371/journal.pcbi.1003482 (PMC3937110; doi:10.1371/journal.pcbi.1003482)
Supplement: Text S2 — Definition of the observables. We define the following observables related to the population structure, as they are relevant for the analysis of the evolutionary dynamics of the social trait: 1) the group size experienced by an average individual of each strategy; 2) the social ratio experienced by an average individual of each strategy; 3) the volatility of each strategy. (PDF) [file pcbi.1003482.s002.pdf]

Text S2: Definition of the observables

Group size

Once groups are determined, an observable of interest is the mean group sizes n_S and n_A experienced by **S** and **A** particles. It is expected that **S**s experience larger groups on average than **A**s owing to their higher attractiveness.

$$n_S = \frac{1}{\mathrm{card}\{j, \sigma(j) = S\}} \sum_{j=1}^{N_{pop}} \mathbb{1}_{\{\sigma(j)=S\}}\, n_j \tag{1}$$

$$n_A = \frac{1}{\mathrm{card}\{j, \sigma(j) = A\}} \sum_{j=1}^{N_{pop}} \mathbb{1}_{\{\sigma(j)=A\}}\, n_j \tag{2}$$

Assortment of strategies

As the individual payoffs depend on the proportion of **S** particles in a group, we are also interested in computing this average social ratio in groups experienced by **S** and **A** focal individuals (keeping only those that are in a group):

$$R_S = \frac{1}{\mathrm{card}\{j, \sigma(j) = S, n_j > 1\}} \sum_{j=1}^{N_{pop}} \mathbb{1}_{\{\sigma(j)=S\}}\, \mathbb{1}_{\{n_j>1\}}\, \frac{s_j}{n_j} \tag{3}$$

$$R_A = \frac{1}{\mathrm{card}\{j, \sigma(j) = A, n_j > 1\}} \sum_{j=1}^{N_{pop}} \mathbb{1}_{\{\sigma(j)=A\}}\, \mathbb{1}_{\{n_j>1\}}\, \frac{s_j}{n_j} \tag{4}$$

Remark: while we discuss qualitatively the spatial segregation between **S** and **A**s within groups in the main text, R_S and R_A are not measures of such spatial assortment. They only account for the average proportion of **S**s found in groups, seen from the point of view of an **S** (R_S) or an **A** (R_S) particle.

Strategy's volatility

The strategy's volatility is defined as the proportion of particles of this strategy that remain alone after the group formation process:

$$u_S = \frac{\mathrm{card}\{j, \sigma(j) = S, n_j = 1\}}{\mathrm{card}\{j, \sigma(j) = S\}} \tag{5}$$

$$u_A = \frac{\mathrm{card}\{j, \sigma(j) = A, n_j = 1\}}{\mathrm{card}\{j, \sigma(j) = A\}} \tag{6}$$
