# Supplementary material for: Differential Adhesion between Moving Particles as a Mechanism for the Evolution of Social Groups
Source: PLoS Comput Biol. 2014 Feb 27;10(2):e1003482. doi: 10.1371/journal.pcbi.1003482 (PMC3937110; doi:10.1371/journal.pcbi.1003482)
Supplement: Text S3 — Details of the evolutionary algorithm used to perform the simulations. (PDF) [file pcbi.1003482.s003.pdf]

Text S3: Details of the evolutionary algorithm used to perform the simulations

The following instructions are repeated at each generation $g = 1, \ldots, g_f$ until an equilibrium is reached:

Initialization

The algorithm is initialized with a population of N_{pop} particles ith positions (x, y) chosen at random on the $[0, L] \times [0, L]$ 2D square, and motion directions θ chosen at random between 0 and 2π.

Aggregation process

At each time step $t = 1, \ldots, t_f$, all particles' updated directions and positions are calculated successively according to the following steps:

1. find the focal particle's neighbors within a radius r_1;
2. calculate the interaction forces they exert on the focal particle according to Equation 1 of the main text;
3. calculate the focal particle's new direction according to Equation 2 of the main text;
4. update the focal particle's position according to Equation 3 of the main text.

Payoff allocation

Once time step $t = t_f$ is over, each particle's group (if any) is determined according to the criterion described in Text S1 of the SI. If the particle j's group has n_j members, among whom s_j are social, then its payoff is calculated according to a linear PGG, that is:

$$P_j = b\,\frac{s_j}{n_j} \qquad \text{if particle } j \text{ is asocial} \tag{1}$$

$$P_j = b\,\frac{s_j}{n_j} - c \qquad \text{if particle } j \text{ is social} \tag{2}$$

We assume clonal reproduction. The payoff received by each individual determines its probability to generate an offspring at the next generation. This probability is linearly determined by rescaling the payoff between two values f_{min} and f_{max} with $0 \leq fmin < fmax < 1$. The rescaling parameters allow to adjust both the speed of renewal of the population and the strength of selection.

Social frequency update

Here, we detail how the frequency $x^{(g+1)}$ of the social trait at generation $g+1$ is determined according to its frequency $x^{(g)}$ and the average probabilities $f_S^{(g)}$ and $f_A^{(g)}$ of an **S** (resp. an **A**) particle to leave offspring at generation g.

Birth process: Let $N_S^{(g)}$ and $N_A^{(g)}$ be the number of **S** (resp. **A**) particles in the population at generation g, so that $N_S^{(g)} + N_A^{(g)} = N_{pop}$. Considering that mutations can arise at a frequency m and change the strategy of a newborn to the opposite strategy (**S** $\rightarrow$ **A** and **A** $\rightarrow$ **S**), their numbers N'_S and N'_A due to the birth process can be expressed as:

$$\begin{aligned} N'_S &= N_S^{(g)} + N_S^{(g)} f_S^{(g)} (1-m) + N_A^{(g)} f_A^{(g)} m \\ &= N_{pop} \left[x^{(g)} + x^{(g)} f_S^{(g)} (1-m) + (1-x^{(g)}) f_A^{(g)} m \right] \end{aligned} \tag{3}$$

and

$$\begin{aligned} N'_A &= N_A^{(g)} + N_A^{(g)} f_A^{(g)} (1-m) + N_S^{(g)} (1+f_S^{(g)}) m \\ &= N_{pop} \left[1 - x^{(g)} + (1-x^{(g)}) f_A^{(g)} (1-m) + x^{(g)} f_S^{(g)} m \right] \end{aligned} \tag{4}$$

Death process: As we want not to take into account demographic effects on the evolutionary dynamics, we maintain the population constant by means of a death process that applies indiscriminately to particles of any strategy. Each particle thus dies with a probability $d^{(g)}$ such that at generation $g+1$ the population size $N_S^{(g+1)} + N_A^{(g+1)} = N_{pop}$. Hence the survival rate $(1-d^{(g)})$ is such that $N_S^{(g+1)} = N'_S(1-d^{(g)})$ and $N_A^{(g+1)} = N'_A(1-d^{(g)})$, and combining these two conditions and Eqs. 3 and 4 we find:

$$1 - d^{(g)} = \frac{1}{1 + x^{(g)} f_S^{(g)} + (1-x^{(g)}) f_A^{(g)}} \tag{5}$$

Thus,

$$N_S^{(g+1)} = N_{pop} \frac{x^{(g)} + x^{(g)} f_S^{(g)} (1-m) + (1-x^{(g)}) f_A^{(g)} m}{1 + x^{(g)} f_S^{(g)} + (1-x^{(g)}) f_A^{(g)}} \tag{6}$$

and

$$x^{(g+1)} = N_S^{(g+1)} / N_{pop} \tag{7}$$
