# Supplementary figures and images for: Differential Adhesion between Moving Particles as a Mechanism for the Evolution of Social Groups
Source: PLoS Comput Biol. 2014 Feb 27;10(2):e1003482. doi: 10.1371/journal.pcbi.1003482 (PMC3937110; doi:10.1371/journal.pcbi.1003482)

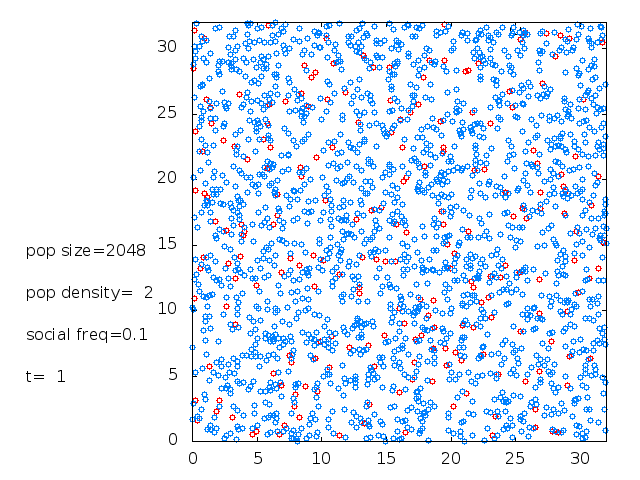

Supplement: Video S1 — Animated GIF displaying an aggregation process. The following parameters are used: . Here the population is mostly asocial and no distinct group forms during the aggregation step. (GIF) [file pcbi.1003482.s004.gif]

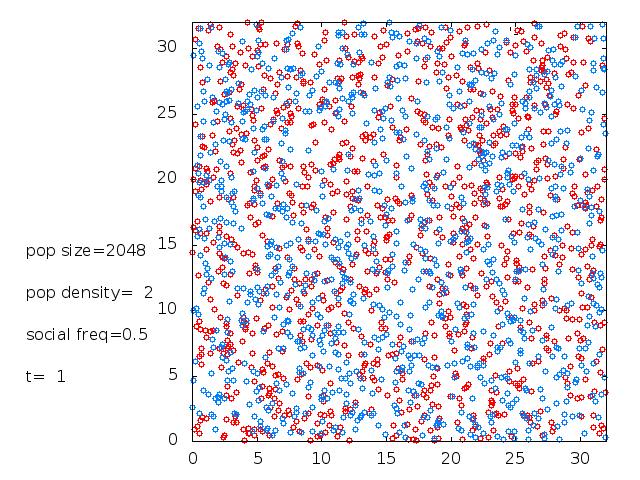

Supplement: Video S2 — Animated GIF displaying an aggregation process. Same parameters as in video S1, except . Here stable groups form during the aggregation step, that are nucleated by a hard core of S particles with A particles at their periphery. A proportion of particles remain alone (not bounded to a group), among those a larger part are asocial. (GIF) [file pcbi.1003482.s005.gif]

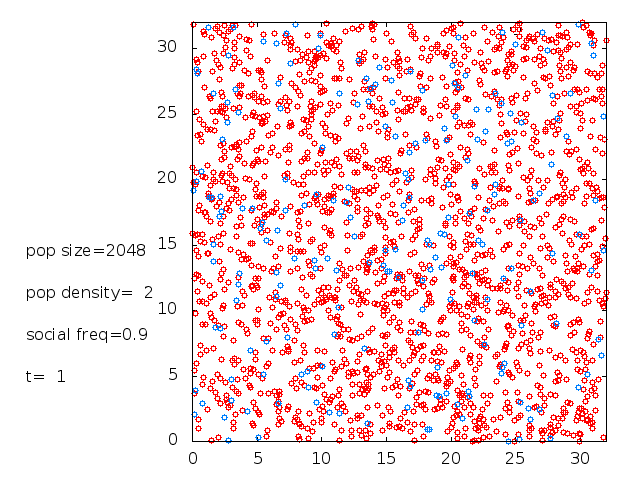

Supplement: Video S3 — Animated GIF displaying an aggregation process. Same parameters as in video S1, except . Here again, stable groups form during the aggregation step, that are on average larger and denser than when . Very few particles remain ungrouped. (GIF) [file pcbi.1003482.s006.gif]
